# Dynamic interactions between a membrane binding protein and lipids induce fluctuating diffusivity

**DOI:** 10.1126/sciadv.1601871

**Published:** 2017-01-20

**Authors:** Eiji Yamamoto, Takuma Akimoto, Antreas C. Kalli, Kenji Yasuoka, Mark S. P. Sansom

**Affiliations:** 1 Graduate School of Science and Technology, Keio University, 3-14-1 Hiyoshi, Kohoku-ku, Yokohama 223-8522, Japan.; 2 Department of Biochemistry, University of Oxford, South Parks Road, Oxford OX1 3QU, UK; 3 Leeds Institute of Cancer and Pathology, School of Medicine, St. James’s University Hospital, University of Leeds, Leeds LS9 7TF, UK; 4 Department of Mechanical Engineering, Keio University, 3-14-1 Hiyoshi, Kohoku-ku, Yokohama 223-8522, Japan.

**Keywords:** Anomalous diffusion, Peripheral membrane proteins, Pleckstrin homology domains, Phosphatidyl-inositol-phosphate, Molecular dynamics simulations

## Abstract

Pleckstrin homology (PH) domains are membrane-binding lipid recognition proteins that interact with phosphatidylinositol phosphate (PIP) molecules in eukaryotic cell membranes. Diffusion of PH domains plays a critical role in biological reactions on membrane surfaces. Although diffusivity can be estimated by long-time measurements, it lacks information on the short-time diffusive nature. We reveal two diffusive properties of a PH domain bound to the surface of a PIP-containing membrane using molecular dynamics simulations. One is fractional Brownian motion, attributed to the motion of the lipids with which the PH domain interacts. The other is temporally fluctuating diffusivity; that is, the short-time diffusivity of the bound protein changes substantially with time. Moreover, the diffusivity for short-time measurements is intrinsically different from that for long-time measurements. This fluctuating diffusivity results from dynamic changes in interactions between the PH domain and PIP molecules. Our results provide evidence that the complexity of protein-lipid interactions plays a crucial role in the diffusion of proteins on biological membrane surfaces. Changes in the diffusivity of PH domains and related membrane-bound proteins may in turn contribute to the formation/dissolution of protein complexes in membranes.

## Teaser

The number of interacting lipids regulates the diffusivity of a peripheral membrane protein bound on a membrane surface.

## INTRODUCTION

Cell membranes provide a unique and complex environment for biological reactions, in which both protein-lipid and protein-protein interactions within the membranes play a key role (*1*–*3*). Diffusion of biomolecules within membranes is crucial for regulating many aspects of cell function. Macromolecular complexity and crowding cause spatiotemporal heterogeneity and thus influence the diffusion process in cell membrane environments.

Peripheral membrane proteins are present within the cytoplasm of cells and associate with cell membrane surfaces in a lipid-dependent fashion. They play key roles in many trafficking and signaling events within cells. Association of peripheral proteins on membrane surfaces is determined by lipid-binding modules, of which the pleckstrin homology (PH) domains are a well-studied family. PH domains are a structurally conserved family of proteins that bind to specific lipids [phosphatidylinositol phosphates (PIPs)] that are present in cell membranes (*4*, *5*). Although structures and membrane interactions have been studied for different PH domains (*6*–*8*), understanding the diffusive behavior of PH domains bound to a cell membrane surface remains challenging (*9*–*11*). Recently, a number of studies have suggested that PIP molecules cluster around membrane-bound peripheral proteins (*12*, *13*). This clustering may affect the diffusivity of peripheral proteins on the membrane surfaces and is thus likely to play a role in regulating their function.

Using single-particle tracking techniques, one can obtain the trajectories of biomolecules. Diffusion is often characterized by the time-averaged mean square displacements (TAMSDs)

$$\bar{\delta^{2}(\Delta ;t)}=\frac{1}{t-\Delta }\int_{0}^{t-\Delta }{\left[\vec{r}(t^{\prime }+\Delta )-\vec{r}(t^{\prime })\right]}^{2}dt^{\prime }$$ (1)

where $\vec{r}(t)$ and Δ(≪*t*) are the position of the tracked particle and the lag time, respectively. In simple diffusion processes, diffusivity is characterized by the slope of the TAMSD for long-time measurements, that is, $\bar{\delta^{2}(\Delta ;t)}\sim 2dD\Delta$, where *d* is the dimension and *D* is the diffusion coefficient. In this case, the diffusion coefficient is uniquely determined depending on the viscosity of the medium and/or the shape of the Brownian particle. However, in living cells, proteins can change their shapes, and properties of the surrounding environments change with time. Therefore, the diffusivity obtained by long-time measurements fails to capture the short-time diffusivity, defined as the diffusivity obtained by short-time measurements, which is considered to be intrinsically fluctuating under diffusion processes. Moreover, anomalous diffusion, seen as a sublinear time dependence of TAMSDs, is not unusual but rather is ubiquitously observed for both proteins in cell membranes (*14*, *15*) and, for example, mRNA (*16*), chromosomal loci (*17*), lipid granules (*18*), and insulin granules (*19*) within cells. Moreover, using molecular dynamics (MD) simulations, subdiffusive motions have also been observed in the diffusion of lipids (*20*–*23*), of transmembrane proteins (*24*), and of water molecules at the surface of membranes (*25*).

Various stochastic models of anomalous diffusion have been proposed to interpret the physical origin of the diffusion process on the assumption that the environment is homogeneous (*26*, *27*). However, this assumption is unlikely to be valid in a cell membrane that presents a heterogeneous environment (*3*). Furthermore, the quenched trap model, that is, diffusion on a random energy landscape, provides a rich behavior that is different from that of a homogeneous environment (*28*, *29*). Revealing the origins of diffusion is important because it will allow us to understand the physical properties of a range of processes, for example, viscoelasticity with crowding of macromolecules (*16*, *18*, *20*–*23*, *25*), transient immobilization in the presence of obstacles with heavy-tailed trapping (*14*, *19*, *25*), and jamming in the presence of obstacles (*14*, *19*). Diffusivity may also change temporally because of changes in the surrounding environment or because of dynamically fluctuating shapes of biomolecules (*30*, *31*). Stochastic models for heterogeneous diffusion processes, in which diffusivity is a spatiotemporally random quantity, have been developed recently in order to interpret the anomalous diffusion of biomolecules in heterogenous crowding environments [for example, spatial heterogeneity (*32*) and temporal heterogeneity (*33*–*38*)].

Here, using MD simulations, we investigate the diffusive behavior of the DAPP1 PH domain on a lipid membrane surface, exploring how the diffusivity of the protein changes with respect to time. Moreover, we show that the fluctuating diffusivity of the bound protein arises from the underlying protein-lipid interactions, which, in turn, dynamically change in time, and that the process is ergodic.

## RESULTS

### Simulations of PH domain interactions with a membrane

**Fig. 1:**
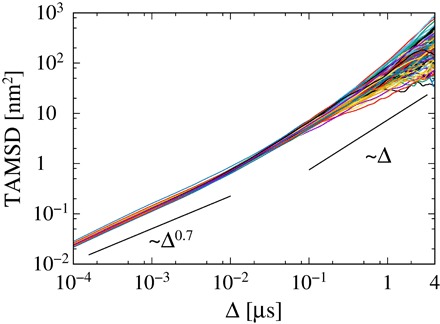
The TAMSDs of 97 trajectories of the PH domain on the membrane surface. The measurement time for each trajectory *t* is 8 μs. The black solid lines are shown for reference.

To investigate the diffusion process of a peripheral protein on a membrane surface, we performed coarse-grained MD (CG-MD) simulations (*39*) of the DAPP1 PH domain interacting with a PIP-containing lipid bilayer membrane. In the initial configuration of each simulation, the PH domain was displaced ca. 9 nm away from the lipid bilayer surface. One hundred simulations were run, each for 10 μs starting from different initial orientations of the PH domain relative to the bilayer, thus yielding a total simulation time of 1 ms. We tracked the protein on the membrane surface and analyzed PH domain diffusion for the last 8 μs of each trajectory for which the protein was bound to the membrane (see fig. S1). Note that 3 trajectories were removed from the initial ensemble of 100 as the protein molecule, having bound, subsequently dissociated from the bilayer. Additionally, we subtracted the center of mass (COM) of the associated bilayer leaflet from the trajectories of the protein to remove effects of COM motion of the membrane as a whole (*20*, *22*, *24*, *25*). The TAMSD of the PH domain on the lipid membrane surface exhibits transient subdiffusion, that is, $\bar{\delta^{2}(\Delta ;t)}\propto \Delta^{\alpha }$ for shorter lag times, switching to$\bar{\delta^{2}(\Delta ;t)}\propto \Delta$ for longer lag times (see Fig. 1). The power-law exponent α changes from 0.7 to 1.0 at a crossover point around 10 ns, which corresponds to the crossover point for anomalous diffusion of lipids within pure lipid bilayers (*20*–*24*). Note that the diffusion coefficient is of the same order of magnitude as the experimentally measured diffusion coefficient of the GRP1 PH domain (*9*, *10*, *13*). Similar transient subdiffusion is also observed for other PH domains (*13*). Moreover, we confirmed that there is no aging of TAMSDs, that is, $\bar{\langle \delta^{2}(\Delta ;t)\rangle }=\;\text{const}˙$, and that there is ergodicity of the diffusion process, that is, the ensemble-averaged MSD is consistent with the TAMSD (see fig. S2).

### Anticorrelated motion of the PH domain over shorter time scales

The diffusive properties of lipids are known to show correlated motions relevant to fractional Brownian motion (FBM) (*20*–*23*). The correlated motions of lipids were also shown to affect the dynamics of interfacial water molecules on the membrane surface (*25*, *40*). To investigate the impact of the FBM of the lipid molecules on the PH domain, we calculated the displacement autocorrelation function (DAF) of the protein

$$C_{\Delta }(t)=\langle (x(t+\Delta )-x(t))(x(\Delta )-x(0))\rangle /\Delta^{2}$$ (2)

**Fig. 2:**
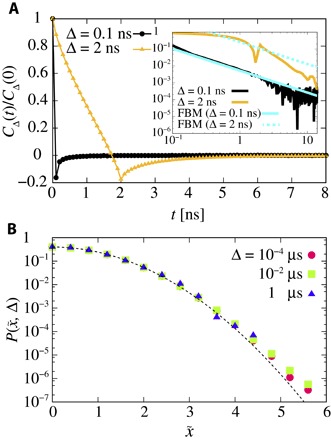
Anticorrelated motion and non-Gaussianity of the PH domain diffusing on the membrane surface. (**A**) Normalized DAF *C*_Δ_(*t*)/*C*_Δ_(0) of the protein for Δ = 0.1 ns (black) and 2 ns (yellow). The inset shows the log-log plot. The solid and dashed cyan lines indicate the theory of FBM. (**B**) The propagator as a function of the normalized position, defined by $x/\sigma \equiv \tilde{x}$, where the SDs σ are 0.1, 0.6, and 5.4 nm for Δ = 10^−4^, 10^−2^, and 1 μs, respectively. Each different symbol represents a different lag time Δ. The dashed line is a Gaussian distribution with unit variance. The propagators deviate from Gaussianity for *x* > 4σ.

Figure 2A shows the normalized DAF *C*_Δ_(*t*)/*C*_Δ_(0) for Δ = 0.1 and 2 ns. The DAF of free FBM decays from negative values to zero via a power law (*41*), *C*_Δ_(*t*)/*C*_Δ_(0) ~ − (α − α^2^)(Δ/*t*)^2 − α^/2. The normalized DAF for Δ = 0.1 ns agrees well with the theoretical behavior of free FBM with α = 0.7 (*41*). Although the normalized DAF for Δ = 2 ns is not consistent with FBM, we can clearly see a cutoff around 10 ns, which implies that TAMSDs correspond to normal diffusion for Δ > 10 ns. The behavior of the protein exhibits anticorrelation comparable to the anticorrelated motions of lipids (*20*–*23*). Thus, the diffusion process of the peripheral PH protein on the membrane surface at shorter time regions is affected by the FBM of lipids with which it interacts.

To confirm whether the diffusive features are well described by the FBM, we examined the Gaussianity of the displacement. In particular, we calculated the propagator, that is, the probability that a particle is found in (*x*, *x* + *dx*) at the lag time Δ. However, the normalized propagator *P*(*x*, Δ) has a non-Gaussian shape (see Fig. 2B). Therefore, the anomalous diffusion of the PH domain may be accounted for by the coexistence with other diffusive properties.

### Fluctuating diffusivity of the PH domain 

**Fig. 3:**
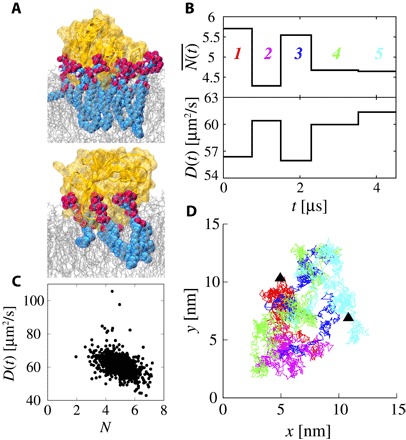
Temporally heterogeneous diffusion process of the PH domain. (**A**) Snapshots of the PH domain in a many-PIP–bound state (upper) and a few-PIP–bound state (lower). The PH domain, lipid bilayer, and bound PIP are colored yellow, silver, and cyan/red, respectively. (**B**) Time series of the short-time diffusivity estimated by our method and the time-averaged number of bound PIPs corresponding to the diffusive state. (**C**) Correlation between the short-time diffusion coefficient and number of bound PIPs in each state. (**D**) Lateral trajectory of a PH domain on the membrane surface. Colors of the trajectory correspond to each state in (B). The black triangles indicate the start and end points.

Interactions between the protein and PIP molecules in the bilayer are crucial for the localization of the PH domain on the membrane surface. As shown in Fig. 3A, the number of PIP molecules interacting with a bound PH domain changes with time. These dynamic interactions are expected to affect the diffusivity of the protein because the diffusivity crucially depends on the properties of the PH/PIP complex, including the number of PIPs present in this complex. In other words, the short-time diffusivity (corresponding to the short-term, that is, sub-nanosecond, subdiffusion regime) may change with time as the number of bound PIPs changes. To better characterize the diffusive behavior of the PH domain, we propose a new method to estimate the short-time diffusivity. To the best of our knowledge, there is currently no method to estimate the short-time diffusivity without knowing the times at which diffusivity changes substantially. Using our estimation method (see Materials and Methods for more details), we can detect variations of the short-time diffusivity as a function of time in a trajectory (see Fig. 3B). Here, we obtained five different diffusive states over the duration of the simulation. Thus, we have successfully detected the fluctuating diffusivity of the PH domain. To investigate the effect of clustering of PIPs about the PH domain on the diffusivity of the protein, we calculated the number of PIPs bound by the PH domain in each diffusive state (see Fig. 3B). We then examined the correlation between the time-dependent diffusivity [*D*(*t*)] and the number of bound PIPs $\left[\bar{N(t)}\right]$ across the whole ensemble of 97 simulations analyzed [see Fig. 3C; the probability density functions (PDFs) of *D*(*t*) and $\bar{N(t)}$ are shown in fig. S3]. It is evident that the diffusivity of the protein is lower when more PIPs are bound than when fewer PIPs are bound. There is a negative correlation (correlation coefficient = −0.42) between the short-time diffusivity and the number of bound PIP molecules. In Fig. 3D, we show the typical trajectory of the PH domain bound to the membrane surface corresponding to Fig. 3B. Although the PH domain diffuses in the same place, the short-time diffusivity differs. This means that the short-time diffusivity of the PH domain undergoes a temporal fluctuation depending not on spatial heterogeneity but instead on temporal changes in the number of bound PIP molecules.

### Heterogeneous diffusion with fluctuating diffusivity

If a system is non-ergodic and/or the time average is not taken for a sufficiently long time period, the TAMSD does not coincide with the MSD. In this case, the TAMSD exhibits broad scattering. The magnitude of the fluctuations of the TAMSD can be quantified by the relative standard deviation (RSD) (*36*–*38*, *42*–*44*)

$$R(t;\Delta )\equiv \frac{\sqrt{\langle {\bar{\delta^{2}(\Delta ;t)}}^{2}\rangle -\langle {\bar{\delta^{2}(\Delta ;t)}\rangle }^{2}}}{\langle \bar{\delta^{2}(\Delta ;t)}\rangle }$$ (3)

**Fig. 4:**
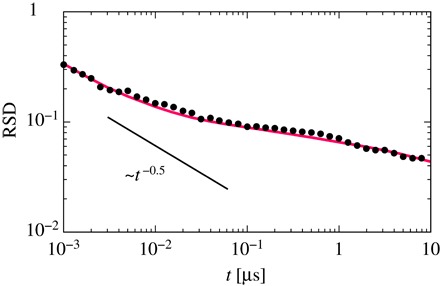
RSD of TAMSDs of the protein (denoted by “• ”). The red solid line represents the RSD of TAMSDs of the LEFD model with γ = 0.6, τ_*c*_ = 10 μs, *D*_*F*_ = 17 μm^2^/s, and *D*_*S*_ = 14 μm^2^/s. The black solid line is shown for reference.

In the case of non-ergodic diffusion processes, for example, the continuous-time random walk (*42*–*44*) and annealed transit time models (*35*), the RSD of TAMSDs converges to a nonzero value for all Δ ≪ *t* as *t* → ∞. This is totally different from ergodic diffusion processes, for example, Brownian motion and FBM in the subdiffusion case (*45*), for which the RSD converges to 0 with a power-law form *t*^−0.5^. In this case, there would be no intrinsic differences between diffusivities for short-time measurements and for long-time measurements. In other words, fluctuations of the TAMSD come from the finite measurement times. However, the difference from the scaling *t*^−0.5^ will imply a possibility that the short-time diffusivity is intrinsically fluctuating. Figure 4 shows the RSD of TAMSDs of the membrane-bound PH domain. The convergence of the RSD to 0 is very slow, that is, the power-law exponent is below −0.5, although the diffusion process is ergodic, as shown by the agreement between the time-averaged and ensemble-averaged MSDs (see fig. S2).

To interpret this, we consider a Langevin equation with fluctuating diffusivity (LEFD) model (*36*–*38*) as a temporally heterogeneous diffusion process, $dx(t)/dt=\sqrt{2D(t)}\omega (t)$, where ω(*t*) is the white Gaussian noise with 〈ω(*t*)〉 = 0, and 〈ω(*t*)ω(*t*′)〉 = δ(*t* − *t*′). The LEFD model is reasonable because the PH domain has been shown to exhibit fast and slow diffusivities depending on the number of bound PIPs (see above), and the diffusion process of this model is ergodic. To capture the essential features of the observed heterogeneous diffusion, we consider the diffusivity *D*(*t*) to vary dichotomously, that is, *D*_*S*_ for a slow state and *D*_*F*_ for a fast state (*D*_*S*_ < *D*_*F*_). From the PDF of the diffusion coefficient, $D=\bar{\delta^{2}(\Delta ;t)}/2d\Delta$ for Δ = 0.1 μs and *t* = 1 μs, we used *D*_*F*_ = 17 and *D*_*S*_ = 14 μm^2^/s (see fig. S4). In the LEFD with a two-state model, sojourn time for each state is a random variable, and we assume that these distributions follow power-law distributions with exponential cutoffs, ρ(τ) ~ τ^−1−γ^ exp (−τ/τ_*c*_), and γ = 0.6 was taken from a previous study (*13*) (see fig. S5), which explored many-PIP–bound and few-PIP–bound states. As can be seen from Fig. 4, the RSD of the LEFD model is surprisingly in agreement with the RSD from the MD simulations of the PH domain (τ_*c*_ = 10 μs was fitted with the RSD of the PH domain). Moreover, the PDF of the diffusion coefficient and the non-Gaussian propagator of the LEFD model are similar to those of the PH domain (see fig. S6). This is further evidence that the short-time diffusivity intrinsically fluctuates with time. In many signaling processes, dynamic interactions of peripheral and integral membrane proteins may be required. Thus, the fluctuating diffusivity of the PH domains and the changes in their local lipid environment may contribute to the dynamics of formation/dissolution of signaling complexes and/or the recruitment/detachment of other peripheral membrane proteins to the membrane.

## DISCUSSION

In summary, we have used extensive (1 ms) MD simulations to investigate the diffusive properties of the DAPP1 PH domain bound to the surface of a model cell membrane. Although the underlying diffusion process is ergodic, the diffusivity of the protein fluctuates anomalously, which may be attributed to the dynamic interaction between the PH domain and the PIP molecules to which it is bound.

Cell membranes are spatially and temporally inhomogeneous environments as a consequence of the formation of lipid (nano)domains, the crowding of a variety of lipid and protein species, and interactions with cytoskeletal components of the cell (*3*). These components of the membrane environment are temporally and spatially regulated. Dynamic interactions of membrane proteins with lipids add a further level of complexity that is therefore expected to make the diffusion process of membrane proteins even more heterogeneous. In our study, we have shown that this heterogeneity determines the diffusive nature of key peripheral proteins on membrane surfaces. In particular, the number of PIP molecules that are bound to a PH domain alters its diffusivity. It is also likely that association/dissociation events may add a further level of complexity to the dynamics of the protein on the membrane surface. However, because we only observed three such events in an ensemble of 100 × 10 μs, we are unable to reliably quantify the consequences of the dissociation/association process. These heterogeneous diffusion processes may be crucial for a variety of biological processes (*46*, *47*). Our results also suggest that lipid molecules not only act as anchors for PH domains but also may provide a mechanism that regulates their function by controlling their diffusion and thus potentially modulating their interactions with other proteins and receptors. It is possible that other classes of peripheral proteins that interact with specific lipids [for example, C2 domains (*48*) and PTEN (*49*)] may exhibit comparable diffusive behavior.

## MATERIALS AND METHODS

### MD simulations

CG-MD simulations were performed using GROMACS 4.5.5 (*50*) with the Martini 2.1 force field (*51*, *52*). The bilayer used in the simulations consisted of POPC (1-palmitoyl-2-oleoyl-*sn*-glycero-3-phosphocholine)/POPS (1-palmitoyl-2-oleoyl-*sn*-glycero-3-phospho-l-serine)/PIP_2_ (phosphatidylinositol 4,5-bisphosphate)/PIP_3_ (phosphatidylinositol 3,4,5-trisphosphate) [259 POPC (73%), 71 POPS (20%), 18 PIP_2_ (5%), and 8 PIP_3_ (2%)] molecules. The systems were solvated with 14,000 CG water molecules, and NaCl ions at a 150 mM concentration were added to neutralize the system. All systems were energy-minimized for 200 steps and equilibrated for 1 ns with the protein backbone particles restrained. For each repeat simulation within an ensemble, the protein was rotated randomly around the *x*, *y*, and *z* axes to form a new initial configuration. For the protein diffusive dynamics, 100 simulations of 10 μs (overall 1 ms of simulation time) with the DAPP1 PH domain [Protein Data Bank code: 1FAO (*53*)] were performed with a time step of 20 fs, and trajectories were saved every 0.1 ns. For each simulation, data for 0 to 2 μs were discarded before collecting data from 2 to 10 μs for analysis of PH domain diffusion. Thus, each simulation analysis starts with a different distribution of PIP molecules around the PH domain (see fig. S7). An elastic network model was applied to all backbone particles within a cutoff distance of 0.7 nm to model secondary and tertiary structures (*54*). The bond lengths were constrained to equilibrium lengths using the LINCS (Linear Constraint Solver) algorithm (*55*). Lennard-Jones interactions were shifted to zero between 0.9 and 1.2 nm, and Coulombic interactions were shifted to zero between 0 and 1.2 nm. A pressure of 1 bar and a temperature of 323 K were controlled using Berendsen’s algorithm (*56*) with a coupling time of 1 ps. The above method predicts the correct DAPP1 PH/bilayer complex, as we have shown in a previous work (*8*). In that work, we have investigated the localization of 13 different PH domains on the surface of a model lipid membrane using a multiscale simulation approach. Strikingly, the PH/PIP complexes obtained by our simulations are similar to the complexes obtained using x-ray crystallography or nuclear magnetic resonance.

### Estimation of short-time diffusivity

Here, we introduced an estimation method to detect the short-time diffusivity. The stages of our method were the following: First, we calculated the TAMSD restricted to the time window [*t*, *t* + *T* − Δ], and thus, we obtained the temporal diffusion coefficients as a function of *t*

$$D(t;\Delta ,T)=\frac{1}{2d\Delta (T-\Delta )}\int_{t}^{t+T-\Delta }{\left[\vec{r}(t^{\prime }+\Delta )-\vec{r}(t^{\prime })\right]}^{2}dt^{\prime }$$ (4)

where *T* is a parameter that we had to determine suitably. Here, we used Δ = 0.1 μs and *T* = 1 μs, which were the same values used for calculating a diffusion coefficient in fig. S3. However, this failed to capture the short-time diffusivity because some time window [*t*, *t* + *T* − Δ] contained the point at which the short-time diffusivity changed substantially. For this reason, we introduced the renewal time at which the diffusivity changed. The renewal time was defined as the temporal diffusion coefficients crossed its mean. We divided the obtained trajectory of *D*(*t*; Δ,*T*) into two states, a fast state *F* and a slow state *S*, using the average calculated by the whole trajectory. The transition point *t*_*i*_ of each state was estimated by *S* → *F* [ρ_*S*_(*t*_*i*_) < ρ_*F*_(*t*_*i*_)] or *F* → *S* [ρ_*F*_(*t*_*i*_) < ρ_*S*_(*t*_*i*_)], where

$$\rho_{F}(t_{i})=\frac{1}{T_{c}}\int_{t_{i}}^{t_{i}+T_{c}}1(D(t^{\prime };\Delta ,T)>D_{a})dt^{\prime }$$ (5)

$$\rho_{S}(t_{i})=\frac{1}{T_{c}}\int_{t_{i}}^{t_{i}+T_{c}}1(D(t^{\prime };\Delta ,T)<D_{a})dt^{\prime }$$ (6)

using the average *D*_*a*_, and we used *T*_*c*_ = 10 ns. The renewal time detected the transition point from a fast to a slow diffusive state or vice versa. Because we knew the renewal times *t*_1_, ⋯, *t*_*i*_, we calculated the short-time diffusion coefficient in the time window [*t*_*i*_, *t*_*i*+1_]

$$D(t)=\frac{1}{2d\Delta (t_{i+1}-t_{i}-\Delta )}\int_{t_{i}}^{t_{i+1}-\Delta }{\left[\vec{r}(t^{\prime }+\Delta )-\vec{r}(t^{\prime })\right]}^{2}dt^{\prime }$$ (7)

where *t*_*i*_ is the *i*th renewal time, and we used Δ = 0.1 ns.

## Supplementary Material

http://advances.sciencemag.org/cgi/content/full/3/1/e1601871/DC1

## SUPPLEMETARY MATERIALS

Supplementary material for this article is available at http://advances.sciencemag.org/cgi/content/full/3/1/e1601871/DC1 fig. S1. Distance between the COM of the protein and the bilayer for the 100 repeat simulations. fig. S2. Ergodicity of the diffusion process. fig. S3. Short-time diffusivity of the PH domain and number of bound PIP molecules. fig. S4. PDF of the diffusion coefficient calculated by $D=\bar{\delta^{2}(\Delta ;t)}/2d\Delta$ for Δ = 0.1 μs and *t* = 1 μs. fig. S5. PDFs of the residence times of many-PIP–bound and few-PIP–bound states. fig. S6. Stochastic simulation of the LEFD model. fig. S7. PIP molecules around the PH domain.
