# Supplementary material for: Dynamic interactions between a membrane binding protein and lipids induce fluctuating diffusivity
Source: Sci Adv. 2017 Jan 20;3(1):e1601871. doi: 10.1126/sciadv.1601871 (PMC5249258; doi:10.1126/sciadv.1601871)
Supplement: http://advances.sciencemag.org/cgi/content/full/3/1/e1601871/DC1 [file supp_3_1_e1601871__index.html]

Science Advances | Science Advances

## Supplementary Materials

**This PDF file includes:**

- fig. S1. Distance between the COM of the protein and the bilayer for the 100 repeat simulations.
- fig. S2. Ergodicity of the diffusion process.
- fig. S3. Short-time diffusivity of the PH domain and number of bound PIP molecules.
- fig. S4. PDF of the diffusion coefficient calculated by *D* = δ2 (Δ; *t*) / 2*d*Δ for Δ = 0.1 μs and *t* = 1 μs.
- fig. S5. PDFs of the residence times of many-PIP–bound and few-PIP–bound states.
- fig. S6. Stochastic simulation of the LEFD model.
- fig. S7. PIP molecules around the PH domain.

Download PDF

**Files in this Data Supplement:**

- Adobe PDF - 1601871\_SM.pdf
