# Supplementary material for: Dynamic interactions between a membrane binding protein and lipids induce fluctuating diffusivity
Source: Sci Adv. 2017 Jan 20;3(1):e1601871. doi: 10.1126/sciadv.1601871 (PMC5249258; doi:10.1126/sciadv.1601871)
Supplement: http://advances.sciencemag.org/cgi/content/full/3/1/e1601871/DC1 [file 1601871_SM.pdf]

Science Advances

AAAS

advances.sciencemag.org/cgi/content/full/3/1/e1601871/DC1

Supplementary Materials for

Dynamic interactions between a membrane binding protein and lipids induce fluctuating diffusivity

Eiji Yamamoto, Takuma Akimoto, Antreas C. Kalli, Kenji Yasuoka, Mark S. P. Sansom

Published 20 January 2017, *Sci. Adv.* **3**, e1601871 (2017)
DOI: 10.1126/sciadv.1601871

This PDF file includes:

- fig. S1. Distance between the COM of the protein and the bilayer for the 100 repeat simulations.
- fig. S2. Ergodicity of the diffusion process.
- fig. S3. Short-time diffusivity of the PH domain and number of bound PIP molecules.
- fig. S4. PDF of the diffusion coefficient calculated by $D = \overline{\delta^2(\Delta;t)} / 2d\Delta$ for Δ = 0.1 μs and t = 1 μs.
- fig. S5. PDFs of the residence times of many-PIP–bound and few-PIP–bound states.
- fig. S6. Stochastic simulation of the LEFD model.
- fig. S7. PIP molecules around the PH domain.

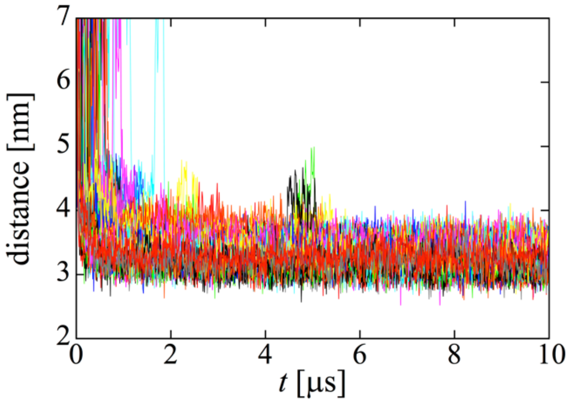

fig. S1. Distance between the center of mass (COM) of the protein and the bilayer for the 100 repeat simulations. In the majority of the simulations the protein binds to the bilayer within the first μs. Thus, we used trajectories for diffusive calculations corresponding to the last 8 μs. We removed three trajectories (light green, black, and yellow) from the ensemble because the distance between the COMs indicated that the PH domain dissociated from the bilayer. A small increase of the value of RSD occurred if we included these three trajectories (not shown).

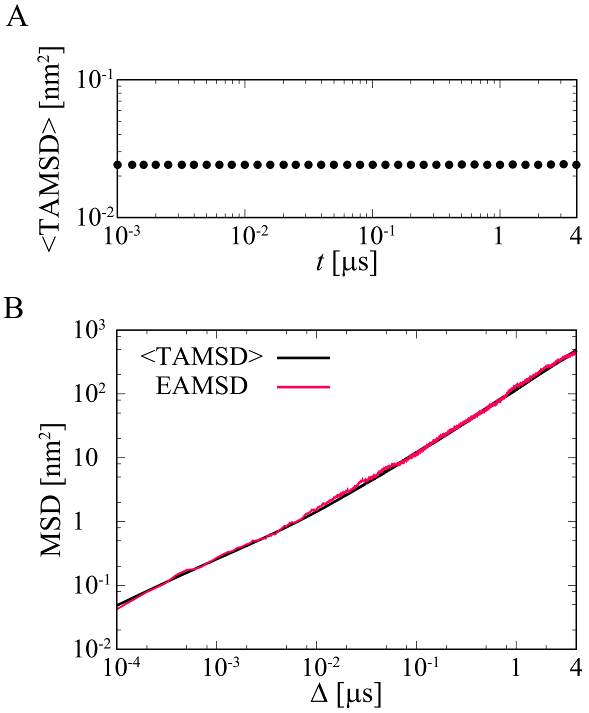

fig. S2. Ergodicity of the diffusion process. (A) Aging plot of the TAMSD, $\langle\overline{\delta^2(\Delta;t)}\rangle$ vs measurement time t. The lag time Δ is fixed at 0.1 ns. These data suggest that there is no aging. (B) Ensemble averaged TAMSD, $\langle\overline{\delta^2(\Delta;t)}\rangle$ ($t = 8$ μs), and ensemble averaged MSD (EAMSD), $\langle\overrightarrow{r}^2(\Delta)\rangle$. Because the TAMSDs converged to the ensemble averaged TAMSD, they are equivalent to EAMSD, i.e. ergodicity.

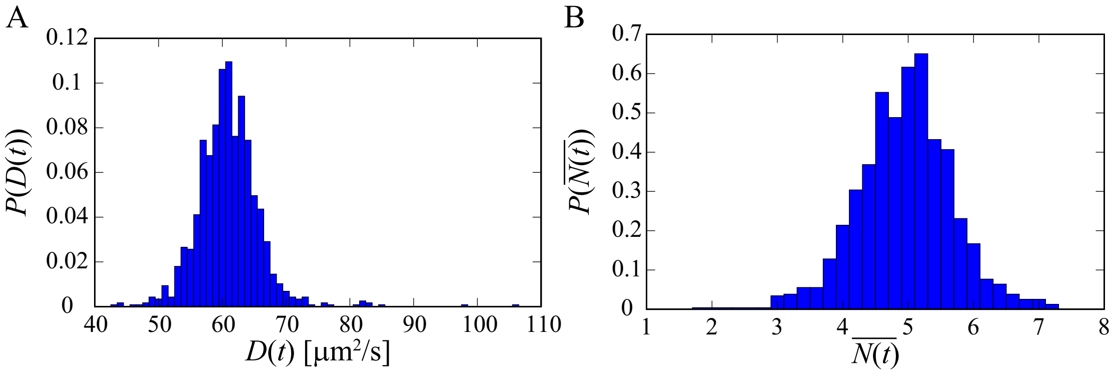

fig. S3. Short-time diffusivity of the PH domain and number of bound PIP molecules.
Probability density functions (PDFs) of (A) the short-time diffusion coefficient $D(t)$ (eq. (7) in the main text) and (B) the time-averaged number $\overline{N(t)}$ of bound PIPs in each state. The lag time Δ is fixed at 0.1 ns.

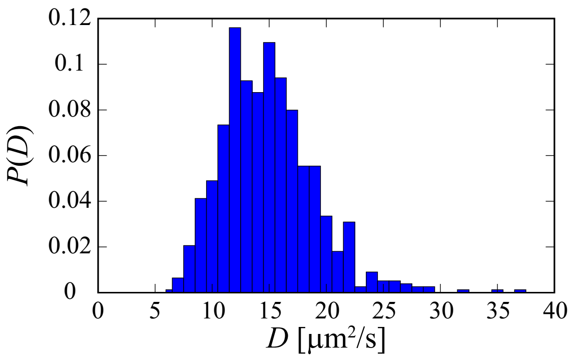

fig. S4. PDF of the diffusion coefficient calculated by D = $\overline{\delta^2(\Delta;t)}/2d\Delta$ for Δ = 0.1 μs and t = 1 μs. We note that Δ is different from that in fig. S3.

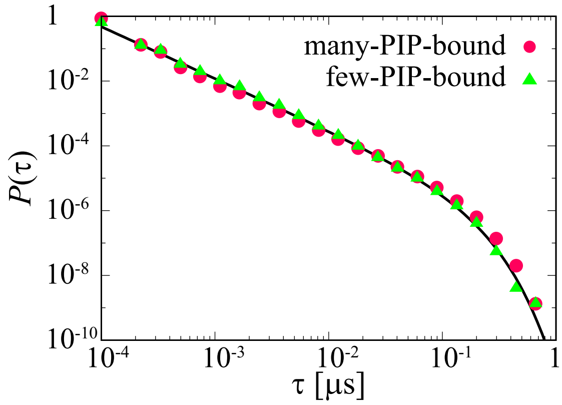

fig. S5. PDFs of the residence times of many-PIP-bound and few-PIP-bound states. The power-law distribution with an exponential cutoff is shown for reference (black solid line): $P(\tau) = A\tau^{-1-\gamma}\exp(-\tau/\tau_c)$, where $\gamma = 0.6$ and $\tau_c = 0.1$ μs.

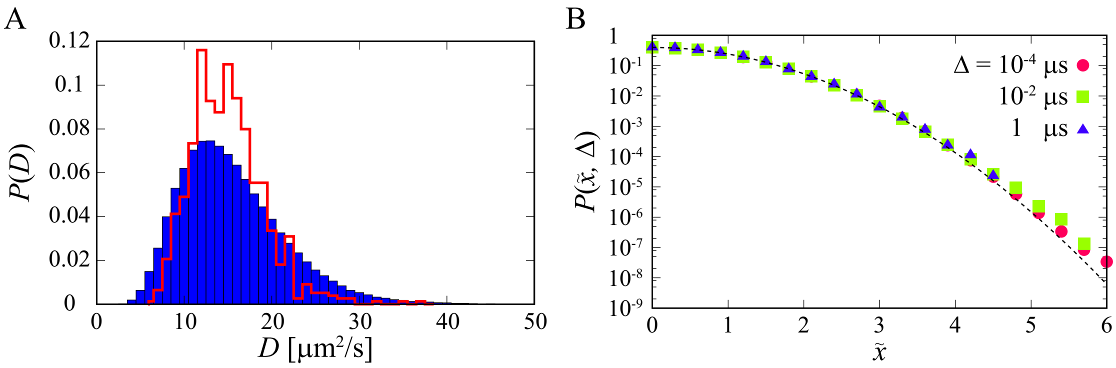

fig. S6. Stochastic simulation of the Langevin equation with fluctuating diffusivity (LEFD) model. (A) PDF of the diffusion coefficient of the LEFD model calculated by $D = \overline{\delta^2(\Delta;t)}/2d\Delta$ for $\Delta = 0.1$ μs and $t = 1$ μs (blue). PDF of the diffusion coefficient of CG-MD is shown for reference (red), where Δ and t are the same as those in fig. S4. (B) The propagator as a function of the normalized position, defined by $x/\sigma \equiv \tilde{x}$. Each different symbol represents a different lag time Δ. The dashed line is a Gaussian distribution with unit variance.

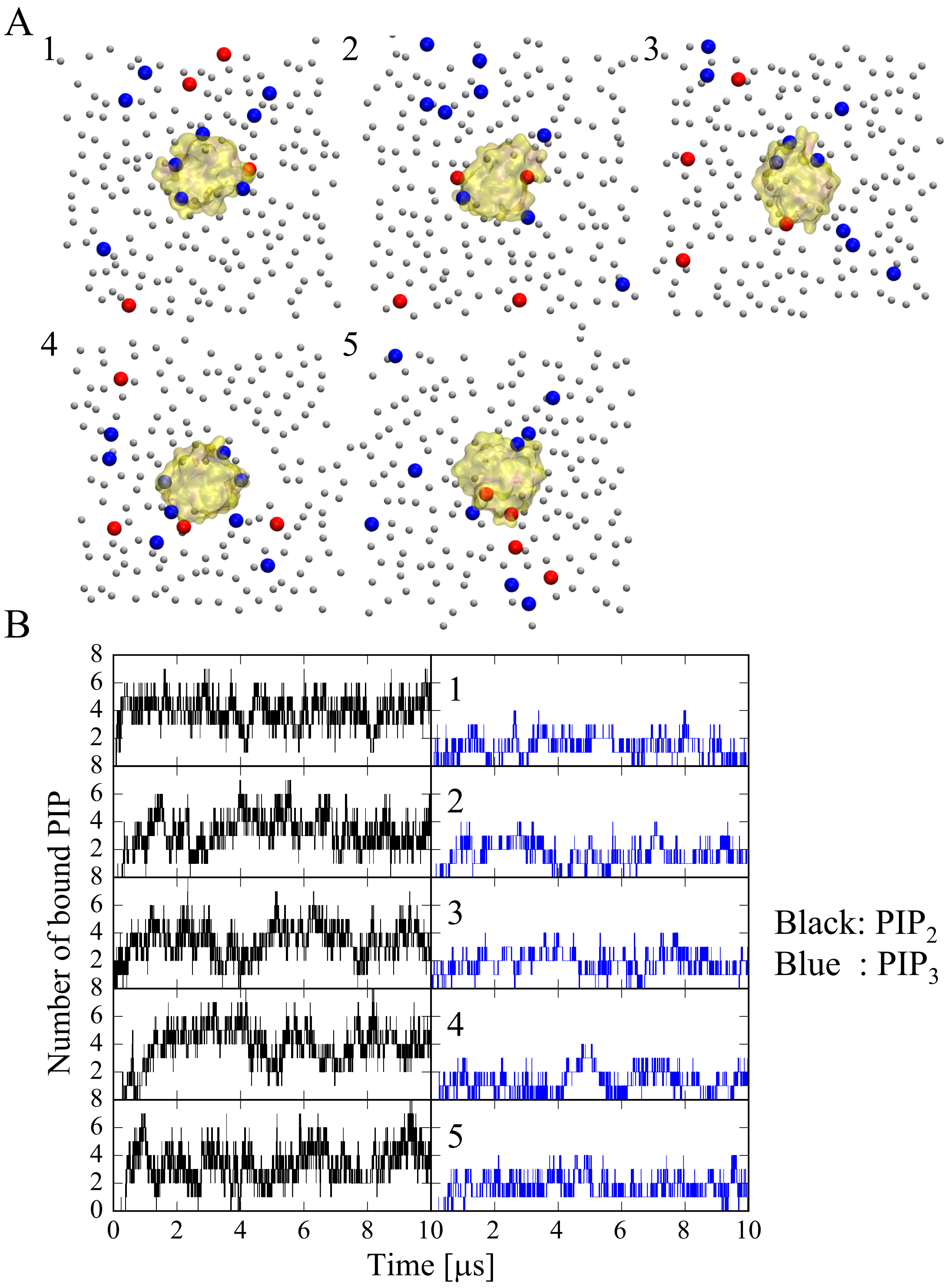

fig. S7. PIP molecules around the PH domain. (A) Five snapshots at time 2 μs of randomly selected members of the ensemble showing the positions of PIP_3 (red) and PIP_2 (blue), of the other lipid molecules (grey), and of the protein (yellow). (B) Number of PIP molecules bound to the PH domain for the same five members of the ensemble as in (A). The black and blue lines correspond to PIP_2 and PIP_3, respectively.
